# Multidimensional Circadian Monitoring by Wearable Biosensors in Parkinson’s Disease

**DOI:** 10.3389/fneur.2018.00157

**Published:** 2018-03-26

**Authors:** Carlos J. Madrid-Navarro, Francisco Escamilla-Sevilla, Adolfo Mínguez-Castellanos, Manuel Campos, Fernando Ruiz-Abellán, Juan A. Madrid, M. A. Rol

**Affiliations:** ^1^Neurology Service, Hospital Universitario Virgen de las Nieves, Granada, Spain; ^2^Instituto de Investigación Biosanitaria ibs.GRANADA, Granada, Spain; ^3^Chronobiology Laboratory, IMIB-Arrixaca, Universidad de Murcia, CIBERFES, Instituto de Salud Carlos III, Murcia, Spain; ^4^Electronics Laboratory, SAI, University of Murcia, Murcia, Spain

**Keywords:** Parkinson’s disease, non-motor symptoms, sleep, wearable, circadian rhythms, wrist temperature, machine learning

## Abstract

Parkinson’s disease (PD) is associated with several non-motor symptoms that may precede the diagnosis and constitute a major source of frailty in this population. The digital era in health care has open up new prospects to move forward from the qualitative and subjective scoring for PD with the use of new wearable biosensors that enable frequent quantitative, reliable, repeatable, and multidimensional measurements to be made with minimal discomfort and inconvenience for patients. A cross-sectional study was conducted to test a wrist-worn device combined with machine-learning processing to detect circadian rhythms of sleep, motor, and autonomic disruption, which can be suitable for the objective and non-invasive evaluation of PD patients. Wrist skin temperature, motor acceleration, time in movement, hand position, light exposure, and sleep rhythms were continuously measured in 12 PD patients and 12 age-matched healthy controls for seven consecutive days using an ambulatory circadian monitoring device (ACM). Our study demonstrates that a multichannel ACM device collects reliable and complementary information from motor (acceleration and time in movement) and common non-motor (sleep and skin temperature rhythms) features frequently disrupted in PD. Acceleration during the daytime (as indicative of motor impairment), time in movement during sleep (representative of fragmented sleep) and their ratio (A/T) are the best indexes to objectively characterize the most common symptoms of PD, allowing for a reliable and easy scoring method to evaluate patients. Chronodisruption score, measured by the integrative algorithm known as the circadian function index is directly linked to a low A/T score. Our work attempts to implement innovative technologies based on wearable, multisensor, objective, and easy-to-use devices, to quantify PD circadian rhythms in huge populations over extended periods of time, while controlling at the same time exposure to exogenous circadian synchronizers.

## Introduction

Advances in sleep and circadian monitoring over the last 20 years have been limited in part by the lack of availability of objective tools capable of quantifying sleep and circadian function in a continuous, simple, and non-invasive manner. The development of wearable multisensor devices and mathematical procedures for big data processing to accurately quantify sleep and circadian disruption (CD) is taking on an important role in personalized medicine by detecting healthy living habits and helping to diagnose and treat several pathologies, including Parkinson’s disease (PD) (1).

Sleep–wake rhythm and circadian system status are currently analyzed by actimetry, combined with specific algorithms to determine the timing and intensity of movements, which are used to infer sleep parameters. This procedure can be useful to detect circadian sleep disorders, but cannot determine sleep and circadian disorders accurately due to the low specificity of actimetry to detect immobile wake states while laying in bed and the high influence of external conditions and volition itself (2). In addition to actimetry, newer techniques being developed include wrist temperature (WT) and light exposure sensors to measure daily fluctuations in sleep propensity, environmental synchronization and autonomic balance (3–5).

While the core body temperature falls before going to sleep and begins to rise in anticipation of waking up, skin temperature increases prior to bedtime and drops just after awakening, in close association with vasomotor skin tone under autonomic control (3–6). In fact, WT combined with actimetry have been validated in both healthy subjects and patients with sleep pathologies against sleep logs (3) and PSG (7), respectively, to determine sleep and CD under normal living conditions. Validation studies have also demonstrated a close association between evening WT increase and dim light melatonin onset (DLMO), suggesting that this rhythm can be a simpler way of measuring circadian phase than melatonin quantification (4). Furthermore, the WT rhythm has a high endogenous component and it is under genetic influence (8, 9), reflects sleep propensity (6, 10), and is also important for the dipping pattern of blood pressure (11).

Parkinson’s disease is a common neurodegenerative disorder characterized by motor symptoms including tremors, rigidity, postural instability, and bradykinesia. However, it is accompanied or preceded by non-motor symptoms that can constitute a major source of frailty in this population (12). Sleep–wake disturbances in PD is one the most frequent and disabling non-motor symptoms (13) and can be secondary to several factors: reemerging motor symptoms during the night, mood disorders, medication, nocturia, parasomnias, and REM sleep behavior disorder (RBD); but it can also be due to direct circadian rhythm disruption caused by the neurodegeneration itself. The suprachiasmatic nucleus seems to be relatively intact in PD, but its neural pathways and the surrounding hypothalamus are more affected (14, 15). Furthermore, in early patients with PD, there is evidence for alterations in melatonin levels and in the expression of molecular clock genes (16). Other signs of circadian impairment in PD are a non-dipping pattern in arterial blood pressure and core body temperature rhythm impairment (17, 18).

This combination of motor and non-motor symptoms, the peculiarity of clinical manifestations for each PD patient, disease evolution and treatment effectiveness assessment make personalization a must, and multisensor devices based on ambulatory circadian monitoring techniques thus constitute a unique tool to bring e-health closer to this group.

Ambulatory circadian monitoring (ACM), a procedure proposed by Ortiz-Tudela et al. (3), is supported by wearable technology which combines four categories of variables useful for tracking complex neurological pathologies such as PD, since:

(1) wrist temperature rhythm is expected to be impaired in PD, as there is an abnormal thermoregulation in the distal skin, with an impaired vasoconstriction response to adrenergic stimulus (19, 20), as well as alterations in normal blood pressure pattern dipping (11).
(2) motor-related variables (integrated acceleration, time in movement and static hand position), indicate both wake states and cardinal motor symptoms of PD disease, and are more dependent on the subject’s habits than they are on the circadian clock. They exhibit a lower genetic influence than the temperature rhythm (8).
(3) hand position variability. This indicates changes in body posture when the patient is lying in bed, which could become impaired along with the evolution of PD.
(4) exposure to light, the main circadian synchronizer (21), exposure can also counteract some circadian and motor symptoms in PD (22).

By combining these major and subrogate variables implemented in a ACM device, clinicians and researchers can have access to an immediate map of motor, autonomic and sleep circadian rhythms, which are useful for improving research, clinical diagnoses and treatment in patients with PD.

Considering how quality of life is affected in PD, there is an urgent need to develop and validate wearable technologies to make e-health available to this population of patients, and objectively track sleep, motor, autonomic disruption, and lifestyle habits. Thus, the aim of this work is to test a wrist-worn device for ACM, intended to personalize the evaluation of the multiple symptoms that manifest in neurodegenerative diseases, such as PD.

## Materials and Methods

### Study Population

A cross-sectional study was undertaken with 24 adult volunteers: 12 patients with PD, who meet the diagnostic criteria according to the UK Brain Bank (PD group) and 12 healthy controls, who match the same demographic characteristics (control group). PD patients were selected by convenience sampling from among those who attended the Movement Disorders Unit of the Hospital Universitario Virgen de las Nieves, Granada (HUVN). Controls were recruited from among healthy non-complainers who were the relatives of students from the University of Murcia. Both groups were encouraged to maintain their normal life style during the week of study and were monitored under free-living conditions. All participants received appropriate information about the study and signed an informed consent form before their inclusion. The study was approved by the Ethics Committee of the University of Murcia and HUVN. All subjects gave written informed consent in accordance with the Declaration of Helsinki. One patient was longitudinally recorded three times, before, 1 week after, and 6 months after starting, using levodopa-carbidopa intestinal gel (LCIG) therapy, an effective treatment for advanced PD.

All patients were treated with l-dopa and/or dopaminergic agonists. Exclusion criteria were: diagnosis of dementia or severe psychiatric co-morbidity, fever, or infection in the previous 2 weeks, current smoking habit or alcohol abuse, diagnosis of diabetes mellitus for ≥10 years or undergoing insulin treatment for ≥5 years, clinical polyneuropathy, endocrinopathies (thyroidopathies or suprarenal gland diseases), arterial disease (Raynaud’s, thoracic outlet syndrome), treatment with medications for excessive daytime sleepiness (i.e., modafinil), treatment with adrenergic agonist/blockers, or a connective tissue disease that could affect skin temperature. None of the patients were shift workers or engaged in transmeridian travel during the previous month. The same exclusion criteria were applied to the control group, in addition to meeting criteria for mood disorders, anything more than mild symptoms on any depression scale and psychopharmacological drugs use.

Trained interviewers assessed the severity of PD according to the Hoehn and Yahr stage. The patients’ clinical disability was assessed according to the Unified Parkinson’s Disease Rating Scale (UPDRS) and subscales. PD patients also completed non-motor and sleep assessments using the second version of the Parkinson’s Disease Sleep Scale (PDSS-2), and the Parkinson’s Disease Questionnaire. Subjects in both groups completed the Pittsburgh Sleep Quality Index (PSQI) and the Epworth Sleepiness Scale. The Levodopa equivalent dose (LED) was determined in PD patients using standardized protocols.

### Ambulatory Circadian Monitoring Device

**Figure 1:**
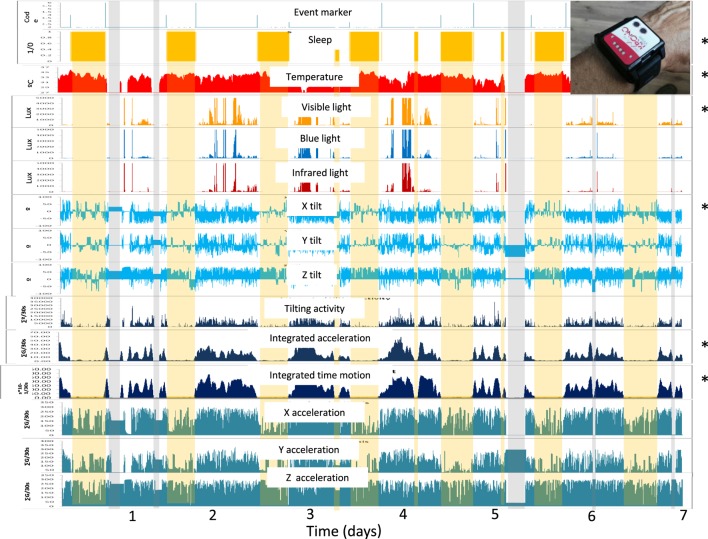
Overview of the fifteen variables recorded during a full week by the ACM device on the wrist of a Parkinson’s disease patient, as processed by Kronoware 10.0 software (Kronohealth SL). From top to bottom: events; sleep (orange bars); skin temperature (red) in °C; visible, blue, and infrared light (orange, blue and red) in luxes; three axis tilt (blue) °/epoch; integrated tilting changes, acceleration and time in movement (dark blue); and partial acceleration of each axis (green). Gray bars indicate “time off” from wearing the device while the yellow bar represents the inferred sleep periods. Asterisks indicate variables selected for the circadian and sleep characterization of Parkinson’s disease patients.

A small, watch-like device for Ambulatory Circadian Monitoring, “Kronowise 3.0” (Kronohealth SL, Spain, Figure 1), was placed on the less affected hand in PD patients or the non-dominant hand in controls, in order to reduce possible masking by motor activity on circadian variables. Wrist skin temperature, triaxial motor acceleration, wrist posture, light exposure in three spectral bands (visible, blue of 460–490 nm and infrared, >800 nm) and an electronic log (event marker) were continuously recorded at 10 (acceleration), 1 (skin temperature and light exposure), or 0.033 Hz (1 reading per epoch) for wrist position. The data were then processed and saved into 30 s epochs for 1 week. A total of 23,000,000 of raw data were internally recorded and processed, and 230,000 of them saved in a txt file for further analysis.

The Kronowise 3.0 device is provided with:

(1) a temperature sensor, with a precision of ± 0.1°C at 25°C and a resolution of 0.0635°C, housed in a separate chamber to avoid thermal interference from the battery and electronic components, with a metal plate in contact with the skin.
(2) A triaxial calibrated MEMS-accelerometer with a linear and equal sensitivity along the three axes, with a range of ± 2 g and a sensitivity of 0.001 g. The *y* axis of the device was aligned with the long axis of the radius; the *x*-axis corresponds to the radial–ulnar axis and the *z*-axis to the palmar–dorsal axis. The default sampling frequency was set at 10 Hz. From the accelerometer output, a total of five groups of motor-related variables were recorded: (a) tilt of the *x, y*, and *z*-axis, as the angle between each axis and the horizontal plane, expressed in °, which allows posture changes to be determined during conditions of immobility (i.e., sleep); (b) the sum of the degrees of change between the current and previous axis position; (c) the area under the curve, integrating the composite acceleration values per epoch; this variable indicates movement velocity and strength, but not the duration or frequency; (d) time in movement, as the cumulative time above a very low threshold (0.05 g) in periods of 0.1 s, in which a movement on any of three axis was detected; (e) the area under the curve for individual *x, y*, and *z* acceleration, in order to discriminate among types of motor activity (i.e., walking, running, typing, etc.).
(3) Three light sensors, on the front, determine visible, infrared, and blue light, with a range of between 0.01 and 43,000 lux, 16 bits of resolution, an internal auto-setting according to the luminance level, and suppression of flicker at 50/60 Hz. The infrared sensor was sensible to radiation from 800 to 1,070 nm, whereas the blue light detector was equipped with a Gaussian filter, which eliminates all visible radiation below 440 and over 500 nm. These wavelengths match the sensitivity of melanopsin retinal ganglion cells (460–480 nm). The infrared/visible light ratio makes it possible to determine the light source (i.e., natural, fluorescent, infrared, incandescent, or LED light).

Communication with the ACM device was established using Kronoware 10.0 software (Kronohealth SL, Spain) *via* a USB port. This software allows visual inspection before analysis to eliminate artifacts and the calculation of basic circadian and sleep parameters. Four calibrated Kronowise devices were used in this study, with minimal differences in recorded variables between them (coefficient of variation < 4%). Data were converted into a text file to be analyzed by the chronobiological software “Circadianware,” implemented on the on-line Kronowizard platform (https://kronowizard.um.es/, University of Murcia).

From the data provided by the ACM device, we selected the following variables (Figure 1):

(a) wrist skin temperature (WT) (as a variable representative of autonomic balance at the skin vessel level).
(b) tilt of the *x*-axis, which oscillates between 0 for maximum horizontality, and 90 for maximum verticality.
(c) acceleration of movement.
(d) time in movement, calculated as the time, in periods of 0.1 s, in which a movement on any of three axes was detected. This information is particularly useful to discriminate between sleep and wake states.
(e) visible light exposure, to determine the intensity and timing of the main synchronizing input to the circadian system.

### Data Analysis

To characterize the circadian pattern, a non-parametric analysis was performed as previously described (3, 6), including:

– interdaily stability (IS) over different days. This varies between 0 for Gaussian noise and 1 for perfect stability, where the rhythm repeats itself exactly, day after day.
– intradaily variability (IV), which indicates rhythm fragmentation; its values oscillate between 0 (when the variable is unfragmented) and 2 (Gaussian noise).
– the mean value and timing of the ten consecutive hours with the lowest values (L10V and L10T, respectively) of WT and sleep probability, and the mean value and timing of the 10 consecutive hours with the highest values (M10V and M10T, respectively) of acceleration, time in movement and light exposure. All these indexes score the extent of activation during the day.
– The mean value and timing of the five consecutive hours with the lowest values (L5V and L5T, respectively) of acceleration, time in movement and light exposure, and the mean value and timing of the five consecutive hours with the highest values (M5V and M5T, respectively) of WT and sleep probability. All these indexes score the restfulness of the sleep period.
– Relative amplitude (RA) refers to the difference between VM10 and VL5, divided by the difference between the two extreme percentiles, Pc95th M10V- Pc5th L5V for acceleration, time in movement and light exposure, with the percentiles extracted from a population of 90 healthy adults enrolled and using the KW3 device (https://kronowizard.um.es/, University of Murcia). The reference values for acceleration were: 40 and 1 for the 95th and 5th percentiles, respectively; 200 and 2 for time in movement; and 3 and 0 for light (log lux). Reference values were rounded to the upper and lower integer for the Pc95th and Pc5th, respectively. Since skin temperature and sleep probability exhibit an inverse pattern to that of motor activity and light exposure, their RA was referred to the difference between M5V and L10V, considering the 95th percentile for M5V and the 5th percentile for L10V (M5V-L10V)/(Pc95th M5V-Pc5th L10V). In this case, the reference values for skin temperature were 35–30° C and 1 and 0 for sleep probability, at the 95th and 5th percentiles, respectively.
– The M10V acceleration/L5V time in movement (A/T) ratio.

### Circadian Function Index (CFI)

Circadian function index was calculated to provide general information about the robustness of the circadian system status of an individual (3). It is computed as the average of IS, IV, and RA, but IV values are previously inverted and normalized between 0 and 1. Thus, a CFI close to 1 indicates a high amplitude, non-fragmented and stable rhythm.

### Sleep Detection

To automatically detect sleep and wake periods, the TAP algorithm (3) was calculated using the Kronowizard website (https://kronowizard.um.es/, University of Murcia). As described by Ortiz-Tudela et al. (3), the TAP algorithm is based on the intra-subject normalization of three signals: wrist skin temperature, time of movement and variability of the *x*-axis tilt per epoch. Since the skin temperature rhythm is the inverse of that for motor activity and position variability, WT was reversed, and thus the maximum of the three daytime variables was considered. The arithmetic mean of the three normalized variables was then calculated in such a way that a 0 value indicated complete rest (sleep), while 1 corresponded to wakefulness and movement. An epoch was scored as sleep when TAP was under a default threshold, previously validated by PSG (7).

Weekly actograms were generated for all variables studied, as well as mean waveforms for every subject and group.

### Statistical Analysis

Data were processed using Microsoft Office Excel 2007. Circadian parameters and PD rating and sleep scales were tested for normal distribution using the Shapiro–Wilk test. All circadian parameters were normally distributed except the ratio A/T. Statistical analyzes (repeated measures ANOVA followed by *post hoc* Bonferroni comparisons for paired samples and correlation analysis using the Pearson’s correlation for normally distributed values and Spearman for not normally distributed values) were performed using SPSS v20.0 (SPSS, Inc. Chicago, IL, USA). Spearman correlations were applied for associations between A/T ratio and CFI score. Bonferroni adjustment was used to set alpha to 0.008 (0.05/6) for multiple comparison correction. Pearson correlations were used to evaluate the association between motor acceleration during daytime (M10V) and PD rating and sleep quality scales. Again, Bonferroni correction was used and alpha set to 0.008. To graphically describe data from PD and control subjects, the Box and Whisker plot method was employed, with the aid of Orange Canvas© software [University of Ljubljana, Slovenia; (23)]. All data were expressed as mean ± SEM.

### Machine-Learning Analysis

All subjects included in our study were classified into PD or C classes using circadian and sleep parameters and by means of machine-learning analysis. This analysis was carried out using the Orange Canvas© software [University of Ljubljana, Slovenia; (23)].

Attribute selection was guided by the expert criterion of including indexes that provide complementary information to one another. Therefore, we aimed to select indexes representative of motor activity and sleep quality. This selection was performed according to the criterion of Information Gain (based on entropy reduction) statistics.

The discretization method used in our study was the Minimum Description Length (24). This is a top-down technique than recursively splits the attribute maximizing information gain, until the point where a new split would not add any new information to the predictions.

The model was evaluated through 10-fold cross-validation, calculating the sensitivity, specificity, accuracy, F1 score and ROC curve for PD discrimination.

## Results

The characteristics of the patients included in the PD group are detailed in Table 1, with ages ranging from 44 to 78 years, and no significant differences in age or gender as compared to the control group. The mean disease duration in the PD group was 12 ± 1.8 years (range of 3–20 years). None of the participants were previously diagnosed with restless legs syndrome or periodic limb movement disorder and only one patient suffered from mild obstructive sleep apnea.

**Table 1:** Participant’s characteristics.

Characteristics	Parkinson’s disease (*n* = 12)	Controls (*n* = 12)
Age (years), mean ± SEM Range	65.83 ± 2.67 54–78	59.41 ± 1.9 (*p* = 0.062) 53–72
Sex (F/M)	3/9	3/9
BMI, mean ± SEM Range	27.26 ± 0.57 24.7–30.8	25.9 ± 0.8 (*p* = 0.31) 20–30.45
Disease duration (years), mean ± SEM Range	12 ± 1.8 3–20	
Levodopa equivalent dose (mg), mean ± SEM Range	1,152.5 ± 134.49 400–1,800	
Hoehn and Yahr (median) stage 2/2.5/3	3/3/6	
UPDRS total, mean ± SEM Range	43 ± 4.65 12–68	
UPDRS II, mean ± SEM Range	9.5 ± 1,41 3–17	
UPDRS III, mean ± SEM Range	25.75 ± 3.18 8–45	
UPDRS IV, man ± SEM Range	5.42 ± 1.28 1–13	
PDQ-39, mean ± SEM Range	48 ± 9.1 11–105	
PDSS-2, mean ± SEM Range	19.27 ± 3.28 3–39	
ESS, mean ± SEM Range	12.1 ± 1.37 4–17	7.17 ± 0.61 (*p* < 0.001) 3–10
PSQI, mean ± SEM Range	7.6 ± 1.18 3–14	5.50 ± 0.54 (*p* = 0.043) 3–8

*F, female; M, male; BMI, body mass index; UPDRS, Unified Parkinson’s Disease Rating Scale and subscales; PDQ-39, Parkinson’s Disease Questionnaire; PDSS-2, Parkinson’s Disease Sleep Scale 2; ESS, Epworth Sleepiness Scale; PSQI, Pitssburgh Sleep Quality Index*.

**Figure 2:**
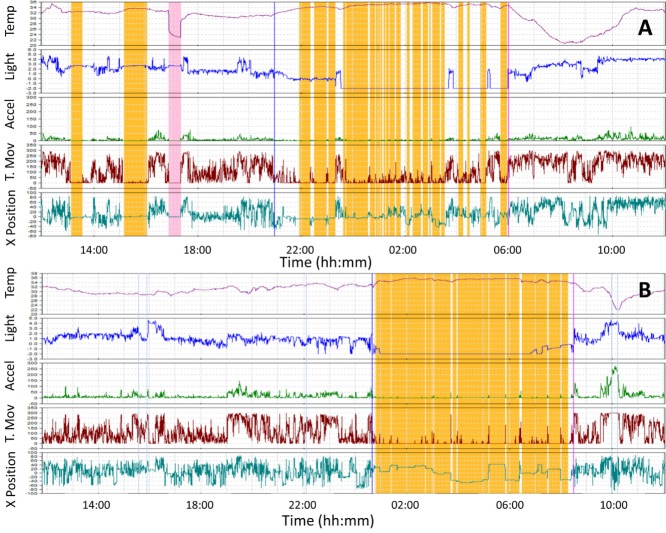
Wrist temperature, light exposure, motor acceleration, time in movement, and 24 h sleep recordings representative for two subjects monitored in our study, one with Parkinson’s disease (PD) **(A)** and a healthy control **(B)**. Note the high nocturnal sleep fragmentation, diurnal sleep, and low motor acceleration of PD. Skin temperature is shown as a red line, visible light in blue, acceleration in green, time in movement in dark red and wrist position (X tilt) in bluish green. Sleep is shown in orange bars. Sensor retrival is indicated by a pink bar.

The ambulatory circadian monitoring device allowed long-term non-invasive recording, with minimal discomfort for the subject. During the 168 days of recording there were no lost data attributable to the device or device removal due to discomfort. From the fifteen variables originally recorded by the device, we selected five, as already stated, for PD characterization, due to their complementarity: skin temperature, acceleration of movement, wrist position, and time in movement (motor symptoms) and exposure to visible light (environmental synchronization). The integration of the information from these five primary variables in the modular TAP algorithm allowed us to infer sleep–wake states (Figure 2).

**Figure 3:**
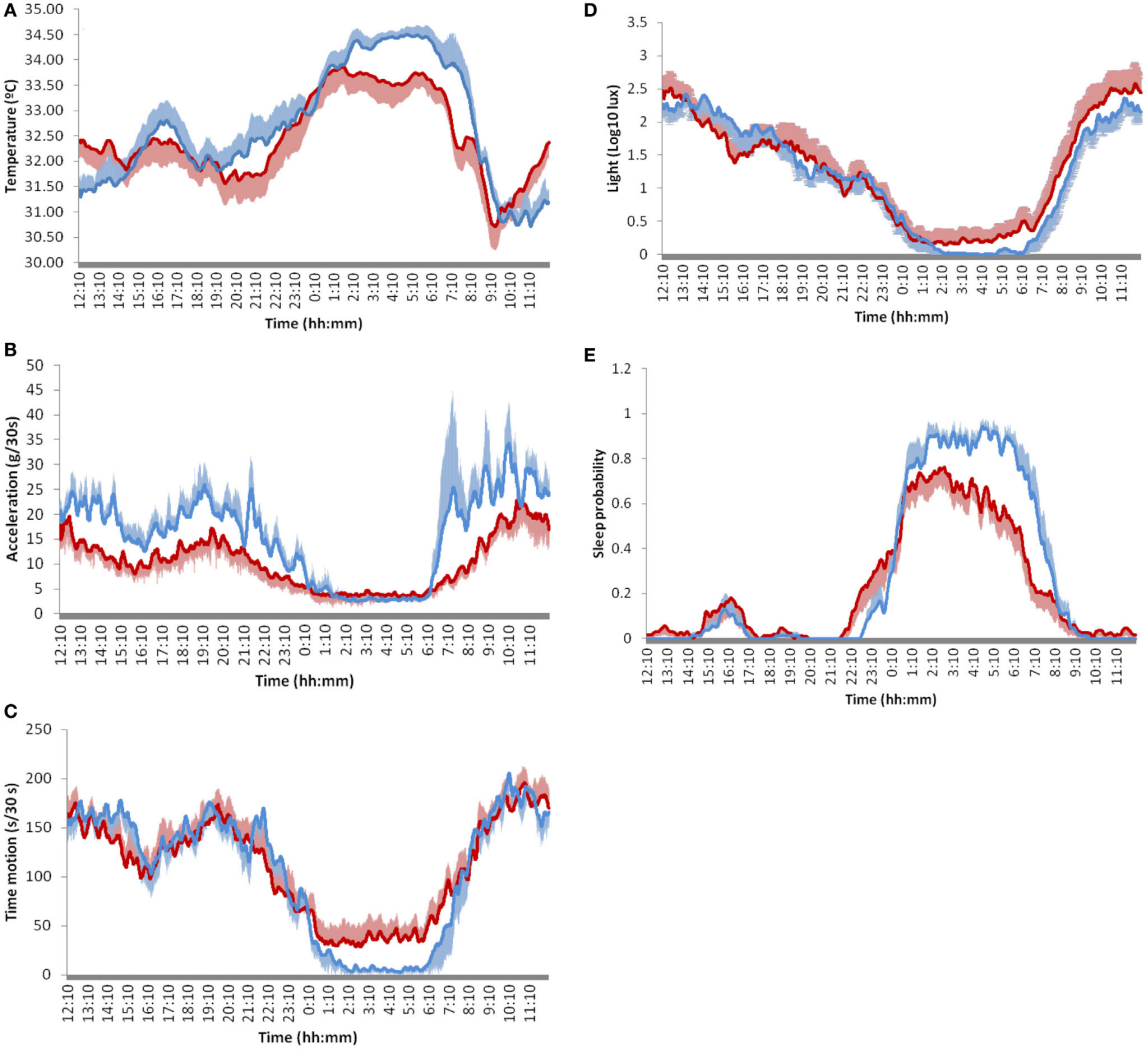
Twenty-four-hour mean waveforms of selected circadian variables in Parkinson’s disease patients (red line) and controls (blue line). **(A)** skin temperature, **(B)** acceleration, **(C)** movement duration, **(D)** light exposure, and **(E)** sleep probability. Values are represented as the mean ± SEM of 12 subjects for each condition recorded every 30 s during 7 days.

As shown in Figure 3, the WT rhythm of healthy controls and PD patients shared common characteristics, which replicated the well-known daily rhythm already described in previous publications (3, 4, 6). In both groups, the WT increases just before bedtime, remains high and relatively stable during sleep and decreases upon awakening, with low and highly variable values during the active phase, and a secondary peak in the afternoon, associated with postprandial somnolence. On the contrary, exposure to light, acceleration and time in movement exhibits an inverse pattern, with lower values as sleep deepens.

However, PD patients show significant differences in all variables (Table 2). They exhibited flattened rhythms as a result of a significant reduction in nocturnal temperature (M5V, *p* = 0.023), sleep probability (M5V, *p* < 0.001) and diurnal acceleration (M10V, *p* < 0.001), together with an increase in nocturnal time in movement (L5V *p* = 0.006). Actual sleep time (not considering sleep latency and wake after sleep onset) was significantly reduced in PD (5:45 ± 0:48 h in PD vs. 6:43 ± 0:24 h in C, *p* = 0.028). In fact, sleep was particularly impaired in the second half of the night, accompanied by early light exposure in the morning and increased motor activity (Figure 3). None of the circadian phase markers differences, including the midpoint of sleep probability (M5T), sleep temperature (M5T), acceleration (L5T), time in movement (L5T), or light exposure (L5T), were statistically significant.

**Table 2:** Circadian parameters representative of the diurnal and nocturnal phases and chronodisruption indexes of the variables selected.

WT			Acceleration		Time in movement		Sleep		Light		Overall	
	Parkinson’s disease (PD)	C	PD	C	PD	C	PD	C	PD	C	PD	C
Mean	32.4 ± 0.23	32.78 ± 0.14	9.39 ± 0.97**	14.58 ± 1.05	102.27 ± 7.61	102.45 ± 4.25	0.24 ± 0.02*	0.28 ± 0.01	1.29 ± 0.15	1.14 ± 0.06	–	–
M5V	33.79 ± 0.24*	34.45 ± 0.14	–	–	–	–	0.72 ± 0.04***	0.91 ± 0.01	–	–	–	–
M5T	3:10 ± 0:30	3:27 ± 0:38	–	–	–	–	3:32 ± 0:27	3:44 ± 0:17	–	–	–	–
L10V	31.68 ± 0.25	31.69 ± 0.24	–	–	–	–	0.03 ± 0.01	0.02 ± 0.01	–	–	–	–
L10T	14:28 ± 0:55	13:32 ± 0:29	–	–	–	–	14:14 ± 0:42	14:57 ± 0:45	–	–	–	–
M10V	–	–	13.55 ± 0.41***	22.59 ± 1.63	146.63 ± 8.67	158.13 ± 6.04	–	–	2:00 ± 0.16	1.98 ± 0.08	–	–
M10T	–	–	14:29 ± 0:25	14:28 ± 0:33	14:51 ± 0:34	14:28 ± 0:35	–	–	14:08 ± 0:19	13:52 ± 0:19	–	–
L5V	–	–	2.95 ± 0.61	2.52 ± 0.41	26.44 ± 7.4**	4.97 ± 0.57	–	–	0.00 ± 0.0	0.00 ± 0.00	–	–
L5T	–	–	4:03 ± 0:25	3:46 ± 0:16	4:02 ± 0:26	3:48 ± 0:16	–	–	3:43 ± 0:26	3:52 ± 0:16	–	–
IS	0.46 ± 0.05	0.55 ± 0.04	0.31 ± 0.03	0.35 ± 0.03	0.41 ± 0.04	0.48 ± 0.02	0.56 ± 0.05***	0.77 ± 0.02	0.57 ± 0.05	0.65 ± 0.03	0.46 ± 0.04*	0.56 ± 0.02
IV	0.003 ± 0.001	0.003 ± 0.001	0.38 ± 0.03*	0.28 ± 0.03	0.27 ± 0.02	0.24 ± 0.01	0.08 ± 0.02**	0.15 ± 0.01	0.08 ± 0.01	0.08 ± 0.01	0.16 ± 0.01	0.15 ± 0.01
RA	0.42 ± 0.04	0.55 ± 0.07	0.28 ± 0.03***	0.50 ± 0.04	0.60 ± 0.05**	0.77 ± 0.03	0.68 ± 0.05***	0.88 ± 0.01	0.60 ± 0.06	0.66 ± 003	0.54 ± 0.02**	0.67 ± 0.03
CFI	0.63 ± 0.03	0.70 ± 0.03	0.47 ± 0.02**	0.57 ± 0.02	0.62 ± 0.03*	0.71 ± 0.02	0.74 ± 0.03**	0.86 ± 0.01	0.71 ± 0.04	0.76 ± 0.02	0.65 ± 0.02**	0.72 ± 0.01
A/T	–	–	–	–	–	–	–	–	–	–	0.72 ± 0.11***	5.48 ± 1.03

Values are the mean ± SEM. WT, wrist temperature; M5V and M5T, mean value and timing of five consecutive hours with the highest values; L10V and L10T, mean value and timing of 10 consecutive hours with the lowest values; M10V and M10T: mean value and timing of 10 consecutive hours with the highest values; L5V and L5T, mean value and timing of five consecutive hours with the lowest values; IS, interdaily stability; IV, intradaily variability; RA, relative amplitude; CFI, circadian function index; A/T, the M10V acceleration/L5V time in movement (A/T) ratio. Level of significance: *p < 0.05; **p < 0.01; ***p < 0.0.

As a measure of chronodisruption, different parameters have been calculated, providing information about complementary aspects characterizing a robust circadian system (Table 2): regularity (IS), day–night contrast (relative amplitude), fragmentation (intradaily variability, IV), and the integrated score CFI. IS was lower in PD as compared to controls, both considering the mean of IS values for all variables (*p* = 0.025) and in particular, for sleep probability (*p* < 0.001). Similarly, day–night contrast was also lower in PD, as indicated by the overall RA mean (*p* = 0.001) as well as by RA for acceleration (*p* < 0.001), time in movement (*p* = 0.007) and sleep probability (*p* = 0.000). Fragmentation (IV) was higher and statistically significantly for acceleration (*p* = 0.014) and sleep (*p* = 0.001) in PD.

Consequently, as a result of the impairment observed in PD in most chronodisruption markers, the integrated CFI score was significantly lower, both overall (*p* = 0.005) and for each particular variable (acceleration *p* = 0.002, time in movement *p* = 0.02, and sleep *p* = 0.001), with the exception of light exposure and WT, as no statistically significant differences were detected in these cases.

**Figure 4:**
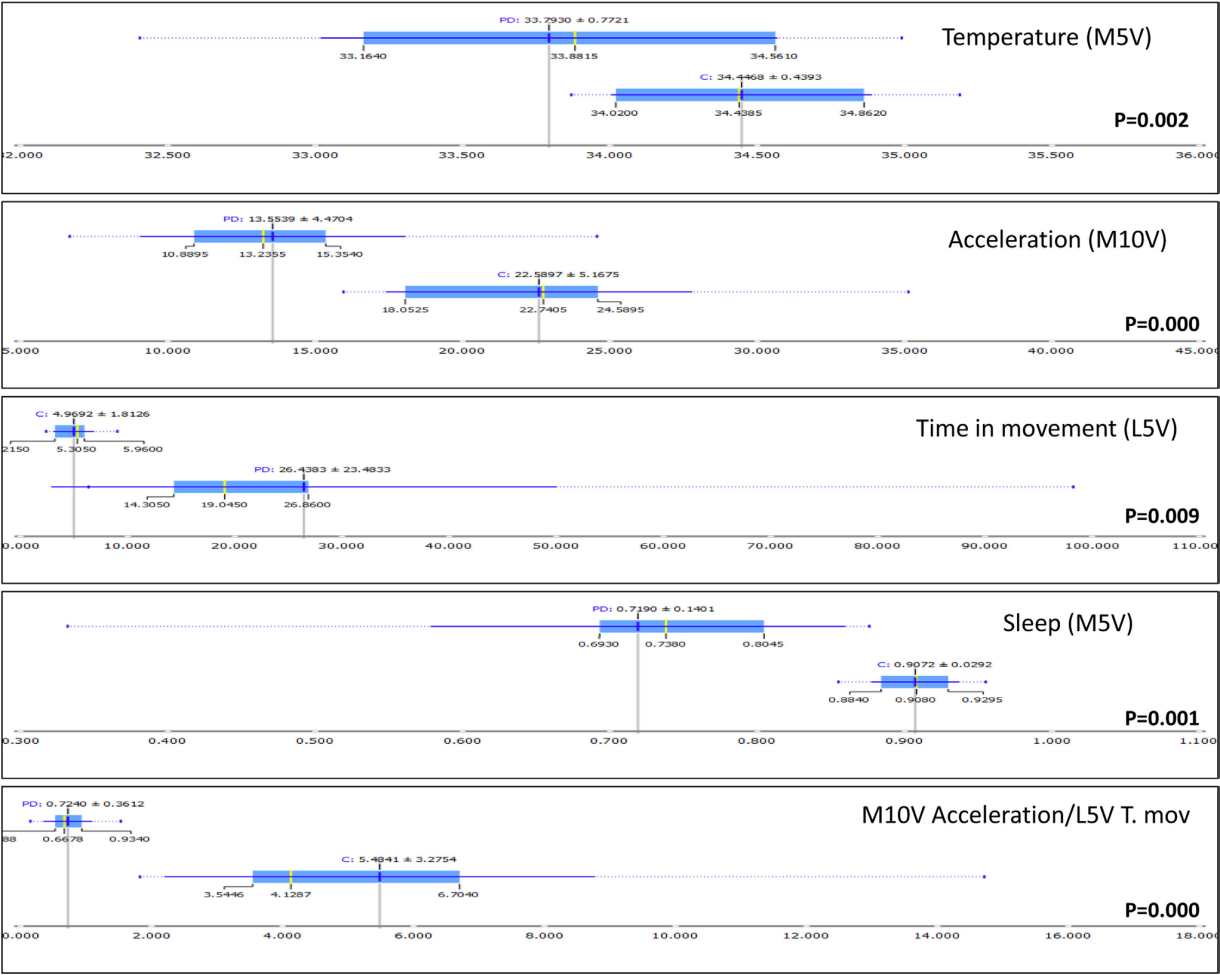
Box plot representation of the distribution of most informative attributes selected according to the information gain procedure (Orange Canvas software) from complementary variables of Parkinson’s disease (PD) and healthy controls (C). The mean values are illustrated by the dark blue vertical line. The blue highlighted area indicates the complete SD of the mean, while the median is represented by a gray vertical line. The thin blue line indicates the area between the first (25%) and the third (75%) quartile, while the thin dotted line represents the entire range of values (from the lowest to the highest value in the data set for the selected parameter).

Thus, ACM provides direct information, allowing discrimination between PD and healthy subjects. Using the Orange Canvas information gain algorithm, the parameters that allow for a better differentiation between PD and control subjects in each variable category were: (a) WT value during sleep (M5V), a reference to autonomic control of skin vasodilatation; (b) daytime acceleration (M10V), since it indicates motor impairment associated with bradykinesia; (c) time in movement during sleep (L5V), a marker of sleep quality and fragmentation; (d) nocturnal sleep (M5V), an index of restfulness; and (e) the M10V acceleration/L5V time in movement (A/T) ratio, which indicates day–night contrast in diurnal activity vs. quiet sleep (Figure 4).

**Figure 5:**
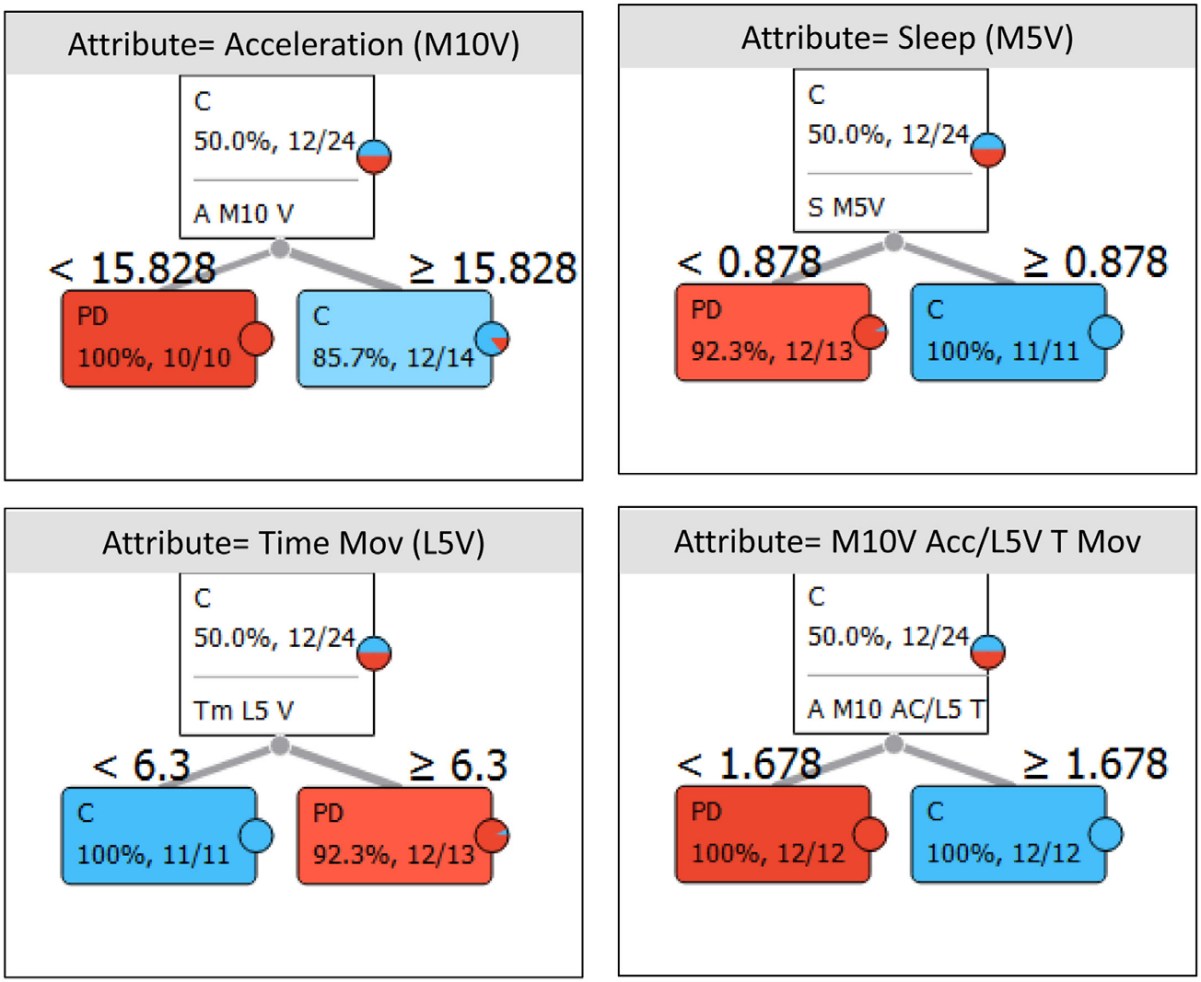
Decision trees for Parkinson’s disease (PD) and healthy controls (C) classification, using those attributes selected from the best discriminating variables and the categorical criteria used for its classification. The number of subjects correctly classified as PD (red box) or C (blue box) in showed inside each box, together with the cut-off point is the upper part of the box.

However, and despite statistically significant differences in M5V for temperature between the PD and control subjects, great variability was observed among PD patients. While some of them exhibited low nocturnal temperatures, others still maintained WT values similar to those of the healthy controls. Much less variability was observed in daytime acceleration or in time of movement during the night. In fact, the cutoff threshold of 15.8 G/30 s for the integrated acceleration and 0.021 s/epoch for time in movement makes it possible to discriminate most PD subjects from the control subjects (only 1 and 2 control subjects were misclassified as PD, using acceleration and time in movement, respectively) (Figure 5). Thus, the M10V acceleration/L5V time in movement (A/T) ratio was ultimately chosen as the best score to differentiate PD from controls and to characterize both motor impairment and sleep disturbance (two of the most common features in PD) based on movements during sleep.

**Figure 6:**
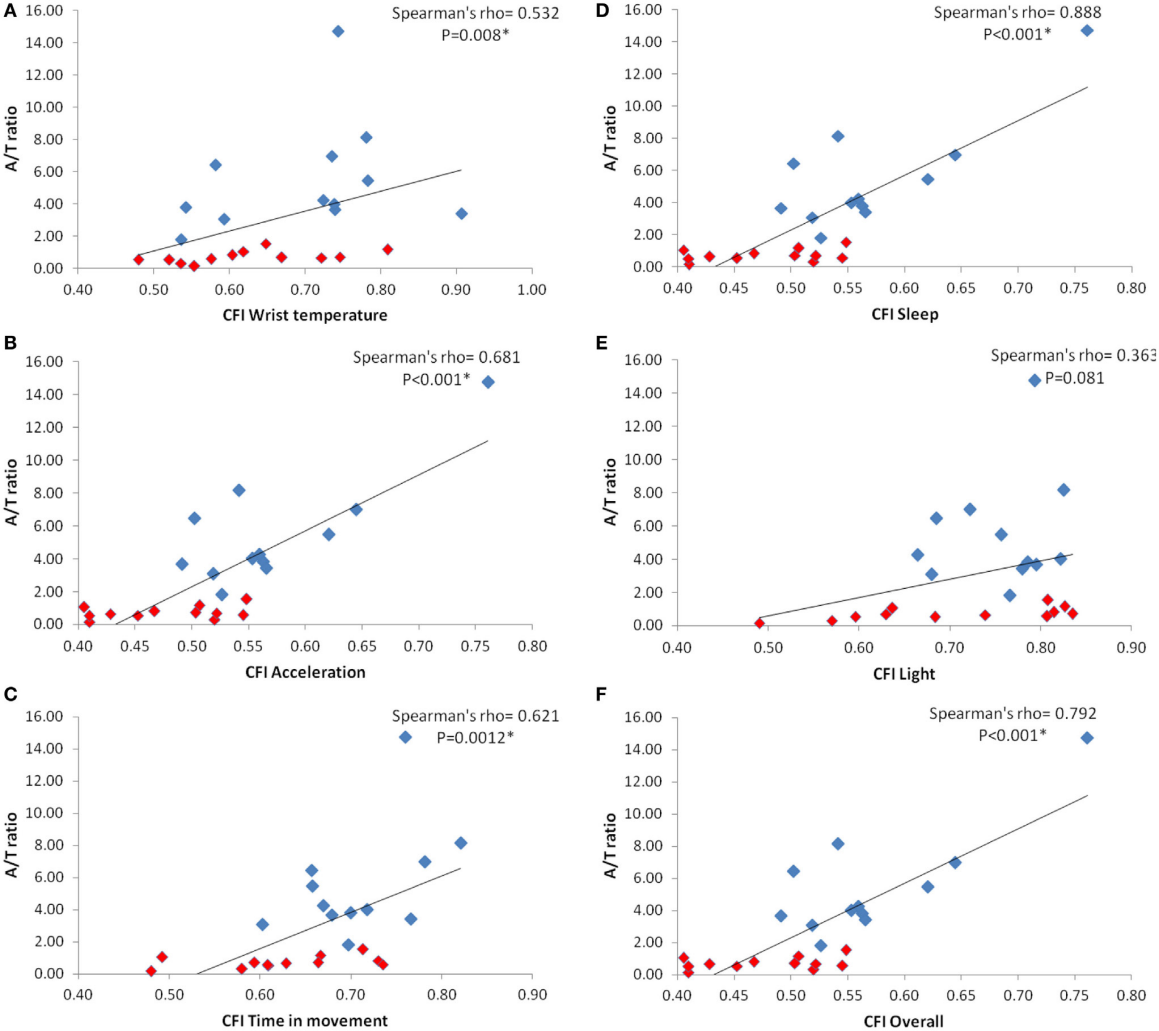
Correlations between M10V Acceleration/L5V Time in movement (A/T ratio) and the circadian function index (CFI), a marker of chronodisruption, for every circadian variable: wrist skin temperature **(A)**; acceleration **(B)**; time in movement **(C)**; sleep **(D)**; light exposure **(E)**; and overall CFI **(F)**. Red squares correspond to Parkinson patients while blue squares indicate healthy controls. Spearman’s correlation coefficient rho and its probability value is shown on the upper right of every panel. * indicates statistical significance after Bonferroni’s correction.

The increase in A/T ratio was linked to a statistically significant higher CFI, a chronodisruption score (ranging between 0, highly chronodisrupted, to 1, a robust circadian system), which integrates in a single value the three main markers of a circadian healthy state, that it is, regularity, fragmentation and rhythm’s amplitude (Figure 6). Thus, CFI of WT (ρ = 0.532, *p* = 0.008), acceleration (ρ = 0.681, *p* < 0,001), time in movement (ρ = 0.621, *p* = 0.0012), sleep (ρ = 0.888, *p* < 0.001) and overall CFI (ρ = 0.792, *p* < 0.001) increased as A/T did, while CFI for light exposure (ρ = 0.363, *p* = 0.081) was not statistically correlated with A/T ratio.

Although the A/T ratio was able to discriminate between subjects with Parkinson’s and controls, and presented good associations with CD markers (CFI), no significant correlations were found between A/T and PD rating scales or subscales (UPDRS, ρ = 0.157, *p* = 0.62; UPDRS II. ρ = −0.19, *p* = 0.55; UPDRS III, ρ = 0.41, *p* = 0.19; UPDRS IV, ρ = −0.34, *p* = 0.28) and sleep quality scores (PDSS, ρ = 0.12, *p* = 0.71; PSQI, ρ = −0.28, *p* = 0.37). However, statistically significant negative relationships were found between acceleration during daytime (M10V) and sleep quality scales (PDSS-2, ρ = −0.71, *p* = 0.008; PSQI, ρ = −0.74, *p* = 0.006). After Bonferroni’s correction for multiple comparisons, no other significant correlations between M10V and PD scales were observed (UPDRS, ρ = −0.59, *p* = 0.046; UPDRS II, ρ = −0.623, *p* = 0.03; UPDRS III, ρ = −0.32, *p* = 0.24; UPDRS IV, ρ = −0.638, *p* = 0.025).

**Figure 7:**
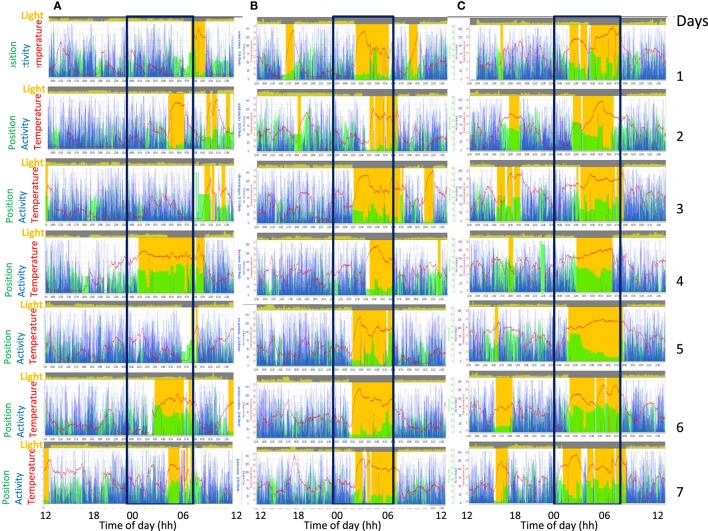
Actograms from ACM recordings of the same patient with advanced Parkinson’s disease, monitorized during 3 weeks: before **(A)**, 1 week **(B)**, and 6 months **(C)** after levodopa-carbidopa intestinal gel (LCIG), treatment. Each recorded day is represented sequentially in the same row. Sleep is shown in orange bars, motor activity in blue, wrist position (X tilt) in green, skin temperature as a red line, and visible light in yellow at the top of each day. Note the progressive improvement of circadian rhythmicity in response to LCIG treatment.

In addition, we recorded the same patient three times throughout the course of the study (Figure 7). A 61-year-old woman with advanced PD was monitored before (Figure 7A), 1 week after (Figure 7B), and 6 months after starting intrajejunal infusion of LCIG (Figure 7C), an advanced therapy to ameliorate her motor symptoms.

The patient experienced an improvement in motor symptoms and in sleep quality. As it can be observed, LCIG therapy diminished the extreme chronodisruption (Figure 7A) characterized by low skin temperature and fragmented sleep and activity rhythms, restoring a more regular, contrasted, and synchronized circadian pattern in all recorded variables (Figure 7B). Once the sleep period was consolidated after the levodopa treatment, sleep time was characterized by an increase in WT, along with a sharp and pronounced decrease in light exposure, acceleration, time in motion and variability in X tilt (Figure 7C). In addition, the position according to the *x*-axis of the device provides relevant information regarding postural changes throughout the night. The A/T ratio increased from 0.15 to 0.75 and 1.99, 1 week and 6 months after the onset of treatment, respectively.

## Discussion

The findings presented here demonstrate the ability of a multichannel ACM device to monitor circadian rhythms and sleep, by collecting reliable and complementary information from motor (acceleration of movement and time in movement) and common non-motor rhythms (sleep and skin temperature) frequently disrupted in PD, with minimal discomfort for patients while they maintain their usual daily living activities. Acceleration during the daytime (indicative of motor impairment), time in movement during sleep (representative of sleep fragmentation) and their ratio (A/T) are the most frequent alterations we have found in PD. Chronodisruption measured by CFI [including IS, intradaily variability (IV) and day-night contrast], are directly linked to a low A/T score. The clinical scales used to evaluate sleep in Parkinson’s patients are also negatively correlated with motor acceleration during the day.

The ACM device complies with all the requirements proposed by the SBSM Guide to Actigraphy Monitoring for actimeters (25), and even goes one step further, overlooking the actigraphic limitations by incorporating new sensors. Thus, ACM integrates new non-invasive measures, validated to predict circadian phase (4, 26), such as wrist skin temperature (WT), and blue, infrared, and full light spectrum. This, combined with the previously validated TAP algorithm, provides reliable information on sleep, circadian timing and chronodisruption.

In fact, WT shows a good correlation with the DLMO (4) and in combination with motor activity and body position, they have been validated by PSG to detect sleep–wake under normal living conditions (7) and in sleep pathologies, such as obstructive sleep apnea (27). The ACM device permits up to fifteen variables to be recorded for 3 weeks in 30-s epochs. From these variables, we selected the five most representative, which have already been validated in healthy subjects: skin temperature, acceleration of movement, time in movement, light exposure, and variability of wrist position. From these variables, a sixth one, sleep probability, was inferred using a TAP algorithm, as previously described (3).

Our results show a flattened circadian pattern in PD patients as compared to the robust rhythmicity detected in healthy controls. This impairment was observed in most recorded variables and could promote a vicious cycle: a disrupted circadian system could contribute to the exacerbation of the clinical symptoms of PD patients and this, in turn, would induce greater chronodisruption (13).

Skin temperature exhibits a well-known circadian pattern determined by an underlying circadian rhythm in thermal regulation and by homeostatic adjustments to environmental and body temperature changes. Since the sympathetic nervous system is the main system responsible for the skin vasomotor changes mediating skin temperature, the impairment of the sympathetic innervation of blood vessels reported in PD (28, 29) could be reflected in the skin temperature rhythm. In fact, our results show that WT decreased in PD during sleep, unlike in healthy subjects, whose temperature reaches maximum levels during the night. However, a great deal of variability was found, since some individuals show very low values in nocturnal and in 24-h mean temperature, while others are in the normal range (although in the lower part) that could reflect variability in sympathetic innervation impairment. Lower temperature during sleep seems to be associated with greater sleep fragmentation, low sleep efficiency, shallow sleep (7), and a non-dipping blood pressure pattern (11); these are also circadian impairments commonly observed in PD patients (30). Sleep disturbances are among the most frequent non-motor symptoms in PD, with an incidence as high as 90% (13). Non-motor symptoms can anticipate the diagnosis of PD by many years (12), thus constituting a possible predictive signal.

Besides changes in skin temperature, sleep timing is also associated in PD patients with elevated nocturnal motor activity time, as has been previously reported (31–33). L5V of time in movement, but not L5V for acceleration, is the most discriminant isolated parameter to differentiate PD patients from healthy subjects. Re-emergent motor symptoms during night, a higher incidence in restless legs syndrome, RBD and nocturia could be responsible for fragmented sleep and longer time in movement in our PD group (13).

By contrast, indexes of diurnal motor activity, such as acceleration integration (M10V), are especially lower in PD patients with respect to the controls. It has been published that diurnal motor activity is flattened overall in association with disease progression (34). These results may reflect the existence of a disrupted circadian rhythm in motor manifestations.

Considering that in our Parkinson’s patients, acceleration, apart from indicating motor symptoms, is most greatly affected during the daytime (with lower values), while time in movement (a marker of sleep quality) increases markedly during sleep time, the A/T ratio contributes to enhancing the differences and facilitates discrimination, constituting an objective score to differentiate PD patients from controls. In fact, reduced nocturnal–diurnal contrast in motor activity with disease severity has previously been reported (33), and a ratio of night-time to daytime motor activity (in acceleration units) has been already proposed to distinguish between controls and PD patients (35). However, the predictive accuracy of this ratio (91.7%) is lower than that of the A/T ratio proposed here (100%). The combination of two complementary methods of measuring motor activity during rest and active phases constitutes, to our knowledge, a significant improvement in scoring the evolution of PD, over a procedure based solely on acceleration. Moreover, the use of this score for a particular patient, before and after LCIG therapy, shows how the disease evolves, in close association with subjective and objective improvements in sleep.

Circadian disruption or significant impairment of the amplitude and synchronization between different rhythms and environmental cues, has been related to a higher incidence and worsening of several pathologies, including metabolic syndrome, cognitive and affective disorders, cancer, accelerated aging, immunodepression, and cardiovascular disease, among others (36). Chronodisruption is common in many neurodegenerative diseases, such as Alzheimer disease and PD, and may contribute by itself to the biology of PD-associated neurodegeneration (17). Our results show that all CD indexes are severely affected in PD, including regularity, fragmentation, day and night contrast and overall circadian system scores, and in a way similar to that observed in the experimental model of Parkinsonism in rats (37).

Coinciding with previous results (17, 32), we confirm an increased intradaily variability in motor activity in PD that can be expanded to other variables, such as sleep, acceleration and time in movement, which presented lower regularity, high IV (an index of the rhythm’s fragmentation, which is also impaired in Alzheimer disease and aging) (38), and amplitude reduction. Reduced amplitude can result from circadian system impairment on three levels: the circadian pacemaker itself, synchronization by input signals or output pathways. Since the suprachiasmatic nucleus appears to be relatively conserved in PD, attention should be paid to input and output pathways. Impairment of anatomical and functional characteristics of the retina have been documented in PD, including dopamine deficiency and impairment of visual acuity and sensitivity contrast (39). Circadian input could be also impaired by inappropriate light-dark exposure, the main circadian zeitgeber; however, we did not find any significant alteration in visible light exposure in PD with respect to healthy controls. In addition to exposure to a regular light–dark cycle, the robustness of the circadian system can be strengthened by consolidated and properly timed behavioral processes, such as physical activity and sleep through feedback mechanisms. In fact, the regularity of life habits facilitates synchronization of the circadian system and is, therefore, considered a protective factor against CD (40). In addition, the fragmentation of sleep in PD patients could likely be responsible for a negative feedback on amplitude and synchronization of rhythms controlled by the central pacemaker and peripheral oscillators.

Output signals from the central pacemaker also appear to be affected in PD, as has been described for melatonin and cortisol secretion (16). Moreover, autonomic skin innervation (41) and distal skin temperature responses are also impaired in PD (20).

It is true that the reduced number of patients in this study makes it difficult to establish general conclusions about circadian rhythms in PD with non-motor symptoms, such as wrist skin temperature and the implication of autonomic vasomotor impairment. However, the main objective of our work was not the validation of a cutoff criteria to discriminate PD from healthy subjects, but to show the viability of a new technology that allows an objective and multidimensional approach to evaluating the symptoms of this disease, in addition to highlighting the heterogeneous character of the symptomatology of PD.

There are others limitations to our study. The patients with PD are very heterogenous with respect to their age and the severity of their illness, which may explain the high variability among patients for some circadian rhythms, such as WT. We did not take into account the differences in anti-Parkinsonism drug treatments or hypnotic medication, which could influence sleep quality in several ways (42, 43). Although only one of our participants was previously diagnosed of obstructive sleep apnea, and none of them presented restless legs syndrome or periodic limbs movement disorder, they were not systematically evaluated by PSG, thus we cannot exclude completely this possibility.

Our work demonstrates the viability of new experimental technologies based on wearable, multisensor and easy-to-use devices that allow a personalized, objective and multidimensional approach to evaluating both motor symptoms and circadian rhythm impairments in PD, which are also valid for other neurodegenerative disorders. Most importantly, these devices make it possible to quantify a large number of participants over extended periods of time, i.e., while treatment takes effect, thus evaluating its effectiveness. Still, large-scale experiments combined with sophisticated signal processing and machine-learning algorithms will be necessary to elucidate whether chronodisruption is a consequence of PD-specific neurodegeneration, or if it can promote the neurodegenerative process of PD.

## Ethics Statement

This study was carried out in accordance with the recommendations of “Ethics Committee of the University of Murcia and HUVN” with written informed consent from all subjects. All subjects gave written informed consent in accordance with the Declaration of Helsinki. The protocol was approved by the “Ethics Committee of the University of Murcia and HUVN.”

## Author Contributions

MR, JM, CM-N, AM-C, and FE-S designed the study and experiments and contributed to drafting the main body of the manuscript. FR-A and JM contributed to electronic design of ACM device. MC is responsible of data processing. CM-N, AM-C, and FE-S managed the subject recruitment and clinical evaluation.

## Conflict of Interest Statement

The authors declare that the research was conducted in the absence of any commercial or financial relationships that could be construed as a potential conflict of interest.
